# Are magnetic resonance imaging or radiographic findings correlated with clinical prognosis in spinal cord neuropathy?

**Published:** 2016

**Authors:** Fatemeh Neshat Halati, Alireza Vajhi, Mohammad Molazem, Mohammad Mehdi Dehghan, Fereshteh Ansari

**Affiliations:** 1 *Department of Radiology and Surgery, Faculty of Veterinary Medicine, University of Tehran, Tehran, Iran;*; 2 *Department of epidemiology, Faculty of Veterinary Medicine, University of Tehran, Tehran, Iran.*

**Keywords:** Dog, Intervertebral disk disease, Magnetic resonance imaging, Treatment

## Abstract

Dogs presented to the Small Animal Hospital of Veterinary Medicine, University of Tehran were included in the present study if spinal or intervertebral disc involvement was suspected. Clinical signs were recorded as well as general information of the patient such as age, breed and sex. Sixty dogs were examined radiographically and two standard orthogonal lateral and ventrodorsal projections were taken from the suspected region. Then magnetic resonance imaging (MRI) was performed for all patients. Agreement between MRI and radiographic findings, comparison of sex and breed with diagnostic imaging grades, comparison between diagnostic imaging grades and mean age, recovery rate after surgery or medical treatment, effects of diagnostic imaging severity grades on surgical or medical referrals were evaluated statistically. There were no significant association between age, sex and breed and frequency of the intervertebral disk disease. Intervertebral disc involvements between L_2_-L_3_ and T_13_-L_1_ were estimated as the most frequent sites of involvements. Sensitivity and specificity of radiography were evaluated 90.0% and 46.0%, respectively, by considering the MRI as a gold standard modality. There was a significant association between severity of disease in the MRI with referral to surgery and medical treatment. The recovery rate after surgery was significantly higher than medical treatment. These results can be used as a foundation for other studies with more focuses on details of injury and larger group of patients.

## Introduction

Neurological deficits related to spinal injuries are common problems in small animal practice that may occur due to various reasons such as inflammatory, anatomical abnormalities, traumatic lesions and intervertebral disc involvements. Intervertebral disc disease (IVDD) is a general descriptive term that is used to define any disc lesions that lead to prolapse of the intact intervertebral disc or nuclear disc material into the vertebral canal and result in compression of spinal cord or nerve roots.^1^ Although IVDD has been classified on the basis of clinical signs, radiographic findings and histologic results, however, magnetic resonance imaging (MRI) is known as the best method for early diagnosis of disc disorders in dogs that is widely available in veterinary medicine nowadays.^2^^-^^5^ According to specific physical features of the MRI, the most complete anatomical investigation can be achieved by this modality and it is known as gold standard evaluation of the spine and spinal cord. Thus, non-invasive detailed examination of the soft tissues such as intervertebral discs, spinal cord, nerve roots and other para spinal anatomical structures may be eased by the mean of MRI.^6^^,^^7^ Classifications of scheme for IVDD and related spinal lesions based on MRI findings have been developed in both human and veterinary medicine but relation of these findings, various severities of the related clinical signs and outcomes after therapeutic management are still uncertain.^8^^,^^9^ Because of the wide availability of MRI for spinal lesions diagnosis, surgical and medical planning in dogs, correlation of the diagnostic imaging findings and followed therapeutic management would be helpful for more reliable prognosis. This study was conducted to answer this question that whether diagnostic imaging findings could be used as a prognostic method to predict recovery rate after surgical or medical treatment.

## Materials and Methods

Sixty dogs which were presented to the Small Animal Hospital of Veterinary Medicine, University of Tehran from January 1, 2012 to January 1, 2014, with chief complaints of back pain, paralysis, fecal incontinence were included in the present study if spinal or intervertebral disc involvement was suspected based on neurological examinations done by an experienced veterinary surgeon. Clinical signs and general information of the patients such as age, breed and sex were recorded.

Included patients were radiographically examined by digital radiography and two standard orthogonal lateral and ventrodorsal projections were taken from the suspected region according to the surgeon’s request. Radiographs were reviewed by two experienced veterinary radiologists independently and results were classified as detectable calcified discs (Group 0), Hansen type II (Group 1) Hansen type I (Group 2), Hansen type III (Group 3) and fractured or luxated vertebra (Group 4). These scores were assigned by authors that are raised from degeneration of disc with intact structure (Hansen II) to annulus fibrosus interruption in Hansen type I and III, and vertebral involvement. It is noticeable that diagnosis of these involvements have been done based on diagnostic imaging signs which are described in elsewhere.^1^^,^^7^

**Fig. 1:**
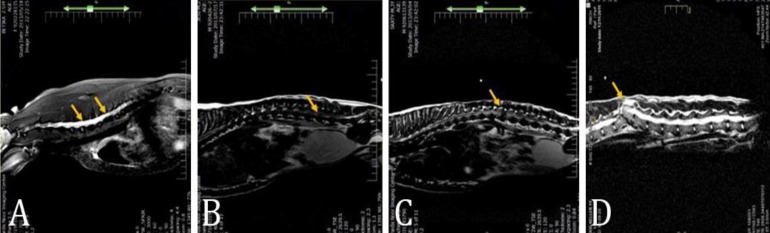
MRI categorization. A) Syringomyelia (Group 2), B) Disc protrusion (Group 3), C) Disc extrusion (Group 4) and D) Vertebral fracture (Group 5

The MRI was performed for all the included patients. Dogs were anesthetized generally with intramuscular 5 mg kg^-1^ ketamine (Alfasan, Woerden, The Netherlands) and 0.08 mg kg^-1^ medetomidine (Syva, Chicago, USA). Magnetic resonance images were obtained by 1.5 tesla magnetic field superconducting magnet equipped with a surface coil and 2 to 3 mm slice thickness in T1 and T2 weighted images. The scanning protocols always included sagittal and transverse planes. The images were evaluated by two veterinary radiologists unaware of the related historical, clinical and radiographic findings using Clear Canvas Workstation software (Version 3.0.16157.49265 SP; Synaptive medical, Toronto, Canada) in order to examining the spinal cord, intervertebral disc, vertebra and presence of protruded or extruded disc materials. Also, MRI results were subcategorized into spinal hemorrhage (Group 0), spinal edema and inflammation (Group 1), syringomyelia (Group 2), disc protrusion (Group 3), disc extrusion (Group 4) and vertebral fracture which was led to disc and spinal involvements (Group 5) and number of the groups used as severity scores (Figs. 1A to 1D). Severity scores in all parts were assigned by the authors based on predictable damage to spine in consequence of etiopathophysiology of each manifestation and also severity of clinical signs in consequence of each involvements.

Clinical reports related to all of the examined patients after one month follow up were extracted from Hospital information system. The results were transformed to “Recovery” where there were reduction in clinical signs, and “no recovery” where regression of clinical signs or no change and increase in clinical signs related to vertebral involvement were seen. All the patients were reexamined after management by the same veterinary surgeon and routine neurological guideline used for scoring and grouping. Clinical signs were recorded neurologically normal (Grade I), ataxia or ambulatory paresis (Grade II), non-ambulatory paresis (Grade III), paralysis with pain perception presence (Grade IV) and paralysis with no pain perception (Grade V).^10^

Generally, agreement between MRI and radiographic findings, comparison of sex and breed with diagnostic imaging grades, comparison between diagnostic imaging grades and mean age, recovery rate after surgery or medical treatment, effects of diagnostic imaging severity grades on surgical or medical referrals were evaluated by SPSS (version 13.0, SPSS, Inc., Chicago, USA).

## Results

A total number of 60 dogs (35 male and 25 female) were included to the present study. Included dogs’ breeds were Mixed Terrier (n = 29), Pekingese (n = 13), Spitz (n = 5), Dachshund (n = 2), Bulldog (n = 2), Shih Tzu terrier (n = 2), Yorkshire terrier (n = 1) and mixed breed dogs (n = 6). The mean age of the patients was six years (ranging from 1 to 11 years).

Only 86.7% of the included patients had suffered from IVDD which include 85.7% of males and 88.0% of females. The patients without IVDD were suffered from calcified discs (n = 5) and fracture or luxation of the vertebra (n = 3).

Intervertebral disc involvements between L_2_-L_3 _(n = 12) and T_13_- L_1_ (n = 8) were estimated as the most frequent sites of involvements (Table 1). Sensitivity and specificity of radiography were evaluated 90.0% and 46.0% respectively, by considering the MRI as a gold standard modality (Table 2).

**Table 1.** Vertebral involvement of extrusion and protrusion

**Intervertebral disk space**	**T**_9_ **- T**_ 10_	**T**_10-_ ** T**_ 11_	**T**_11-_ ** T**_ 12_	**T**_12-_ ** T**_ 13_	**T**_13-_ **L**_1_	**L**_1-_ **L**_2_	**L**_2-_ ** L**_ 3_	**L**_3-_ ** L**_ 4_	**L**_4-_ ** L**_ 5_	**L**_5-_ ** L**_ 6_	**L**_6-_ ** L**_ 7_	**L**_7_ **-S**_1_	**Total**
**Protrusion**	1	1	1	2	3	2	3	1	2	0	1	2	19
**Extrusion**	0	0	3	4	5	4	9	3	3	2	0	0	33
**Total**	1	1	4	6	8	6	12	4	5	2	1	2	52

**Table 2:** Sensitivity and specificity of radiography

**Parameters**	**Result of radiology**		**Total**
	**Positive**	**Negative**	
**Negative on MRI**	8	7	15
**Positive on MRI**	41	4	45
**Total**	49	11	60

The cases without intervertebral disc involvements had 100% recovery rate. Evaluation of the recovery rate revealed that 100% of patients which suffered from hemorrhage, edema, syringomyelia and fracture were recovered. Only 71.4% and 96.3% of the patient which suffered from disc protrusion and extrusion, respectively, were recovered. Recovery rate was estimated 91.4% in this study regardless of type of management. Regression in clinical signs was seen in 5.1% of the patients. No statistical association was detected between rate of recovery and MRI severity scores in cases which were suffered from intervertebral disc involvements, however, slight increased recovery rate were observed by increasing the severity in these cases.

Fisher test showed that the recovery rate after surgery is significantly higher than in medical treatment (p < 0.05), (Table 3). The result showed 21.4% clinical signs regression of the cases were suffered from disc protrusion.

**Table 3:** Recovery from medical treatment and surgery

**Parameters**		**Outcome (**%**)**		**Total**
		**No recovery**	**Recovery**	
**Treatment**	**Medical**	5.0	8.0	13.0
		38.5	61.5	100
	**Surgery**	2.0	29.0	31.0
		6.4	93.6	100
**Total**		7.0	37.0	44.0
14.3%	85.7%	100%

## Discussion

Despite the available scientific definition of the “prognosis”, definition of the successful managing procedure or results vary between both surgeons and owners as it is obvious in different studies.^11^ We can variously define successful management from severity reduction of the clinical signs to returning complete normal ambulation. During the current study we had a hypothesis to find a relationship between diagnostic imaging criteria and prognosis for guiding the surgeons to make the best decision. Overall, we found no statistical association between severity of MRI findings and rate of the recovery, although the 100% recovery of the cases without intervertebral disc involvement and slight increased recovery rate in higher scores of intervertebral discs involvements could be clinically of importance.

Sixty dogs were evaluated during this study with neurological clinical signs. Although there were no statistical associations between breed and involvement to IVDD, the most commonly affected breed in this study was the mixed Terrier, which was different from previous studies.^12^^-^^14^ This may be due to different availability and people’s tendency to adapt various breeds in our country. The mean age of the dogs in this study was six years, similar to previous studies.^15^^,^^16^ In spite of no significant statistical difference between the affected males and females, males were more than female in the present study that is comparable with other studies by Ferreira et al. and Penning et al. ^11^^,^^15^

The results of the present study showed that the most frequent intervertebral disc disease was between L_2_-L_3_ and T_13_-L_1_. However, T_13_-L_1_ was also reported as the second most common affected region in previous studies,^17^^-^^19^ L_2_-L_3_ predisposition can be added to high risk side of IVDD occurrence.

Significant association between MRI finding and referral rate to surgery shows that higher severity in MRI is tend to be referred to surgery by the surgeon. However, it may have an effect on the higher rate of recovery in our study. It is predictable that medical treatment is not promising to eliminate the underlying disease.

We found regression of the clinical signs after treatment of disc protrusions. The rate of this regression was equal to the percentage of the patients which were suffered from disc protrusion and were referred for medical treatment.

The first and major limitation of the present study was that the owners of the severe cases with negative deep pain asked for euthanasia, therefore, they were not included in our study and we should accept that it could have effects on our results. The surgeons and owners were in charge of making decision of final treatment procedure and it could influence the results. At last, it would be helpful to input the clinical grading to our comparisons additionally.

In conclusion, we did not find significant relationship between diagnostic imaging findings and recovery rate and a significant correlation between severity of disease in the MRI and referral to surgery was detected. The significant higher rate of recovery than medical treatments may show the importance of the surgical approaches in severe cases. These results can be used as a foundation for other studies with more focuses on details of injury and larger group of patients without bais of the surgeon or owners attempt for choosing the management approaches.
